# Night-Time Light Data: A Good Proxy Measure for Economic Activity?

**DOI:** 10.1371/journal.pone.0139779

**Published:** 2015-10-23

**Authors:** Charlotta Mellander, José Lobo, Kevin Stolarick, Zara Matheson

**Affiliations:** 1 Jönköping International Business School, Jönköping University, Jönköping, Sweden; 2 School of Human Evolution and Social Change, Arizona State University, Tempe, United States of America; 3 Inclusive Design Research Centre, OCAD University, Toronto, Canada; 4 Former Martin Prosperity Institute, Rotman School of Management, University of Toronto, Toronto, Canada; University California Los Angeles, UNITED STATES

## Abstract

Much research has suggested that night-time light (NTL) can be used as a proxy for a number of variables, including urbanization, density, and economic growth. As governments around the world either collect census data infrequently or are scaling back the amount of detail collected, alternate sources of population and economic information like NTL are being considered. But, just how close is the statistical relationship between NTL and economic activity at a fine-grained geographical level? This paper uses a combination of correlation analysis and geographically weighted regressions in order to examine if light can function as a proxy for economic activities at a finer level. We use a fine-grained geo-coded residential and industrial full sample micro-data set for Sweden, and match it with both radiance and saturated light emissions. We find that the correlation between NTL and economic activity is strong enough to make it a relatively good proxy for population and establishment density, but the correlation is weaker in relation to wages. In general, we find a stronger relation between light and density values, than with light and total values. We also find a closer connection between radiance light and economic activity, than with saturated light. Further, we find the link between light and economic activity, especially estimated by wages, to be slightly overestimated in large urban areas and underestimated in rural areas.

## Introduction

Homo sapiens is now an urban species with over half the world’s population living in urban areas, including many millions in informal settlements [1]. Urbanization is a hallmark of the 21^st^ century, characterized by massive demographic shifts and an unprecedented rapid expansion of urban areas and the built environment [2]. Our planet has indeed become a “planet of cities” [3]. (Throughout the text we use the terms “cities”, “urban areas”, and “metropolitan areas” interchangeably, avoiding the unnecessary controversy of defining them and relying on the readers’ sense of what differentiates urban from non-urban population agglomerations.) The pressing challenges of sustainability, adaptation to climate change, economic recovery and poverty reduction are in effect, all urban challenges. Studying urban dynamics at a truly global scale is therefore an urgent research task, but also one which is hampered by the absence of comprehensive, consistently-defined, and reliably collected data on urban economic output, population size and physical presence. This lamentable empirical situation is not surprising given the inherent difficulties in collecting socio-economic and demographic data at the sub-national level, the typically low frequency of detailed national data collection, and the scaling back of national household data collection in places like the U.S. and Canada. The urban databases with an international scope which do exist—such Moody’s Analytics’ “Global Metro Areas” (www.economy.com/globalmetros), the OECD’s Metropolitan Database (www.oecd.org) and the Brookings Institution’s Global MetroMonitor (www.brookings.edu)—leave much of the developing world uncovered and are constructed mainly by imputation from national-level relationships to assign values to variables meant to capture urban characteristics.

It is difficult to imagine how a scientific understanding of urbanization can be developed without global data on the processes and consequences of urbanization. Even the simple, yet analytically essential, exercise of comparing urban economic performance across regions and nations is currently impaired by the absence of data on urban GDP. In the midst of this empirical sterility, one type of data has recently come to be seen by urbanists as providing the means to overcome the scarcity of global urban information (e.g. [4; 5]): the Nighttime light (NTL) data from the U.S. Air Force’s Defense Meteorological Satellite Program/Operational Linescan System (DMSP/OLS). A hallmark of contemporary urban settlements, and of urban activity in general, is the artificial illumination of buildings, transportation infrastructure (such as roads, airplane runways and railway lines), parking lots, and other components of the built environment. Succinctly stated, wherever humans agglomerate, there will be artificial light. The DMSP satellites, in low sun-synchronous polar orbits, generate a global night time and day time coverage of the Earth every 24 hours with the main purpose of monitoring the distribution of clouds and assessing navigation conditions [6]. The U.S. Department of Commerce’s NOAA National Geophysical Data Center (NGDC) takes the data from the DMSP satellites and, after extensive manipulation, puts it through several algorithms to produce an annual database of night-time lights emissions [7]. A digital archive of the data is available starting with the year 1992 (For data documentation and download, go to: http://ngdc.noaa.gov/eog/).

While the brightness and spatial extent of anthropogenic visible near-infrared emissions (i.e., night-time lights) obviously depend on a variety of socioeconomic and cultural factors [8], and although many non-urban phenomena generate night-time light—including burning agricultural fields, fishing vessels, natural gas flares, and natural and human-made fires—these data have been extensively used by researchers as a measure of urbanization. The global reach of NTL data, and the consistent manner in which it has been collected, make it a unique dataset with which to study urbanization at a planetary scale. Specifically, NTL data has been deployed to identify the real extent of urban agglomerations and to estimate urban population size [6, 7; 9; 10; 11] as well as population density [12]. These data have also been used to track the pace and type of urbanization [13; 14], and measure electricity use, energy consumption and greenhouse gas emissions [15]. More ambitiously NTL data has been used as a proxy indicator of national, regional and urban Gross Domestic Product [4; 5; 6; 16; 17; 18; 19]. With regards to capturing economic activity, the general result from these and other studies is that night-time lights do provide a robust indicator of such activity albeit the relationship seems to be statistically stronger for developing economies than developed ones. In the developed economies the services sector, which are less reliant on physical infrastructure, account for a greater proportion of overall economic output and artificial light generation might exhibit decreasing elasticity of demand as income levels rise.

The strong empirical link between urban habitation and the generation of artificial light seems straightforwardly, even self-evidently, robust. And to the extent that the principal source of energy used for generating artificial light is electricity, so is the use of NTL data as a proxy measure for urban electricity consumption—this can even be stretched so that night-time light emissions are used as an indicator of overall energy use. The connection between the generation of artificial light and economic activity, however, seems to us to require more exploration than has been proffered so far, especially at a finer spatial level. While NTL data can capture the emissions from lampposts at a factory’s parking lot, the satellites cannot detect light use inside a cavernous production plant, whether mostly empty or running at full capacity. Nor can the satellite’s sensors pick up the light emitted from offices in buildings, or distinguish between buildings with offices staffed by high-valued software developers and one crowded with textile workers. But how close is the relationship between NTL emissions and economic activity at a micro-level? And is it mainly related to day-time industry activities or an effect of night-time residential location patterns and structures? As to this point, no one has investigated the direct link between economic activity at the micro-level and the generation of night-time light emissions. What is needed is a fine-grained examination, both at the level of economic units and spatial resolution, of the association between economic activity and the generation of artificial light. This will allow us to gauge just how sensitive an empirical probe night time lights really are. If the national-level results pointing to a strong relationship between economic activity and artificial night-time lights are to be interpreted as more than aggregation effects it is necessary to understand the artificial light intensity of socio-economic activity at the micro-level. We take it as a given that night-time light data is a proxy measure for human agglomerations—the question is just how good of a proxy is it for day-time economic activity?

Here we examine the link between economic activity and NTL emissions by taking advantage of the fine grained spatial resolution at which NTL data is available and combining it with economic data from Sweden that is also available at a very detailed spatial resolution. The NTL data is provided in a grid of 30 arc-seconds projected on the globe, while the Statistics Sweden data assumes a flat projection of Sweden. At the latitude of Stockholm (59.3294° N), each NTL “square” is 472.5m east-to-west by 926.1m north-to-south. The Statistics Sweden grid is in squares either 1,000m by 1,000m or 250m by 250m depending on the underlying population. Statistics Sweden (the Swedish government agency responsible for producing official statistics) has collected geo-coded data for all individuals and establishments (a single physical location at which business is conducted and/or services are provided.) on a yearly basis since 1985 (www.scb.se). By matching NTL data with the Swedish data we can examine just how close the correlation is between specific levels of economic activity and specific levels of NTL emissions. Our a priori assumption is that these variables are correlated but the research questions address how closely correlated they are at a micro-level, and whether information about the relationship between NTL emission and economic activity is clearly differentiated from that contained in the relationship between NTL emissions and population size. We emphasize that we are not seeking to develop yet another method to derive a measure of GDP from data on artificial light emissions, but rather to identify the strength of the empirically and analytically prior relationship between level of economic activity and NTL emissions. Neither is our focus on the circumstances under which NTL is a better or worse predictor of often hard to observe economic or population data. The focus of our investigation is subtly different. NTL data has been used in the literature as a proxy for socioeconomic variables and as a proxy measure for economic output. We seek to explore—availing ourselves of fine-grained observations—whether NTL emissions are actually highly correlated with economic activity. Our chosen analytical focus bears on the econometric framework used to obtain the results presented here. While this inquiry could be reformulated as an investigation of how well NTL emissions predicts other socioeconomic variables we are more interested in the strength of the association between NTL and widely used measures of economic activity, and to what extent this varies across regions.

The detail and spatial resolution of the Swedish socio-economic data, down to 250 by 250 meters, makes it a very valuable analytical resource, but how relevant or applicable are insights gleaned from such data? Sweden is, after all, a fairly small country population-wise, with slightly less than ten million inhabitants, whose large land area (almost 70% that of France and 25% larger than that of Germany) yields a low population density (roughly equal to that of the United States but 11 times smaller than that of Germany). Sweden’s historical peculiarities and its relative isolation from many of the social cross-currents that have shaped modern Europe, as well as its long-standing commitment to social-democratic egalitarianism must also be considered. Is Sweden just too different from other developed economies for Swedish findings to be useful?

In many ways, however, Sweden is typical of the “advanced” European economies. Sweden has been a member of the European Union for almost two decades, and many of the country’s policies and regulations have been brought into alignment with the EU’s framework [20]. The country’s economy is heavily oriented toward foreign trade, with privately owned firms generating the vast majority of economic output and the high-technology engineering sector accounting for about 50% of total output and exports [21]. Sweden’s GDP per capita (41,000 in Purchasing Power Parity 2012 dollars) ranks above that of Germany and South Korea, and slightly below that of the United States. What about Sweden’s energy consumption? Based on data provided by the Department of Energy’s Energy Information Administration (www.eia.gov), Sweden’s energy use is fairly typical of an advanced industrial economy (controlling for its Northern location and its use of energy for heating purposes). The energy intensity per capita (for the time period 2005–2009) in Sweden averaged 245, which could be compared with the US (328), Germany (328), South Korea (199), or Brazil (62). For energy use measured in 2005 purchasing power adjusted in dollars, Sweden measures 7,065, while the US has a value of 7,625, Germany 5,503, and South Korea 9,959. We have, in other words, no reason to believe that Sweden should be over or under-consuming in terms of energy. We therefore believe that examining the micro-level relationship between economic activity and the generation of night-time light data in Sweden will provide insights applicable to other wealthy, highly developed, technologically advanced, and export-oriented market economies.

Although Sweden is an example of a developed (“wealthy”) economy, it is important to notice that wealth and economic activity still do vary among regions inside the country. There are three metropolitan regions—Stockholm, Gothenburg and Malmo—that are relatively large agglomerations that account for a large share of Sweden’s economic activity. At the same time, the country also has a strong mining industry in the northern parts of the country (around Kiruna), were we would expect large economic values per capita from a day-time perspective, while the night-time, residential values would be significantly lower. There are also major differences between urban and rural parts of the country, both in terms of day-time and night-time activities. Rural areas in Sweden are for the most part declining in population. There is also a relatively big divide in industrial activities, where most of the service-based activities are located in urban centers, while rural areas are more manufacturing and resource based industries. In other words, the relatively large geographical area of Sweden covers a large variation in both industrial as well as residential patterns. From earlier research [8] we know that systematic non-urban activities (e.g., from gas and fishing industry) can affect the NTL emissions. While Sweden exhibits little or none of these activities, they or similar effects would be captured by local variation in our geographically weighted regressions. Further, Sweden covers a wide range of longitudes, which provides the opportunity to examine for any potential influence of longitude on the relationship between light emissions and industrial and residential economic activity. Again, any variation in the relations caused by longitudinal differences should be captured by the geographically weighted regressions, since this technique generates local estimates.

The discussion is organized as follows. The next section describes the night-time lights emission data and the Swedish data, as well as the matching and merger of these two variables. The results are presented in section three, with a conclusion in section four. Anticipating our main results, we find that night-time light emissions are a better proxy for population density than for total population, and that the radiance light is slightly more closely related to people and establishments than the saturated light. We also find that the relation between light and economic activity (especially in terms of wages) is underestimated in rural areas and small and medium sized cities, while it is overestimated in the largest regions. The relatively weak relationship between night-time light and economic activity suggests that light is a restricted proxy for economic activity at a micro-level. At the same time, the relatively strong relation to population density suggests that it is a good proxy for understanding urbanization across regions around the world.

## Data Sets and Variables

### Night-Time Lights Data

We used Nighttime Lights Data made available by the U.S. National Oceanographic and Atmospheric Administration (NOAA). The observations on which the data is assembled are made by the Operational Linescan System (OLS) flown on the Defense Meteorological Satellite Program (DMSP) satellites [22]. An excellent detailed description of the satellite instrumentation, and the data collection and processing methods is provided by Elvidge, et al. [23]. Here we restrict ourselves to a brief summary. The DMSP program is designed to capture information about global weather and weather systems. It needs to provide up-to-date daily information. Cloud cover and weather systems can be identified and analyzed visually at any point during the daytime. However, clouds are difficult to spot at night. To that end, the satellites (there are two of them) have onboard sensors designed to detect moonlight (and even starlight) that is reflected off of clouds. Of course, when the night is clear, no clouds get in the way, and the instrument detects the light emanating from the surface of the Earth. That light, as previously mentioned, can be from several sources but is primarily the result of electricity-powered illumination.

Unfortunately the data from the satellite OLS instruments has a high gain filter applied on the ground in the visible light band for the specific purpose of cloud detection. (“Gain” is a measurement unit for how much light per unit is detected by a photomultiplier (light intensifier instrument) of the sort used by the DMSP satellites.) There is an implicit tradeoff: while the satellites’ instrumentation can detect even dim lighting present at the Earth's surface—emanating from small settlements all the way up to Las Vegas or gas [6]—the recorded data are saturated in the bright cores of urban centers. Saturation is due to a limitation in the sensor itself. The sensor is only able to record light up to a reported value of 63 (with zero being the absence of light). All remote sensing products derived from the OLS sensor have this limitation. So, in effect, the intensity of light is not measured past a certain threshold value: the light emitted at the center and much of the surrounding area of say New York, Mumbai, or Tokyo is quantified to be at the same level as that emitted in their peripheries. Saturation occurs in almost all cities of any significant size across the globe. Letu et al. [24] discuss the saturation problem in depth and present one possible method of addressing it. In 2006 permission was granted by the DMSP to readjust the calibration for selected periods of time so that human-generated night-time light emissions (NTL data) could be detected and not suffer from the saturation problem. The process by which the 2006 “radiance” NTL data, as it is known in the remote sensing research community, was constructed is discussed in Ziskin et al. [25]. (Radiance data, and associated documentation, is available at ngdc.noaa.gov/eog/.) As of the end of 2011, the radiance calibrated data, which is a conversion from the original digital numbers to physical units, provides the most accurate and available record of night-time light emissions from human settlements but, to emphasize, it is available for only one time period (in contrast to the “standard” NTL data electronic records which are available yearly starting in 1992).

NTL data is provided in 30 arc-second grids through geo-referenced TIFF images and covers most of the world (the polar areas are excluded). The grid essentially puts down squares on a globe. But since it is a (rough) sphere and not a cube, the grid creates what essentially looks like squares at/near the equator, but as the measurements move towards the poles, the “squares” become narrower at the pole ends. This effect increases the closer you are to either the North or South Poles. The data provided does not include the polar extremes because of both measurement and projection issues.

We employ two different datasets in our analysis: “Global Radiance Calibrated Nighttime Lights” based on satellite readings collected in 2006 (referred to as “Radiance Light” in this discussion), and the standard “Average Lights x Pct” (which we refer to “Saturated Light”) also based on satellite readings from the year 2006. We also used two other NTL datasets, the “Average Visible” and “Stable Lights, & Cloud Free Coverage” for the year 2006 (www.ngdc.noaa.gov/dmsp/downloadV4composites.html). This data performed very similarly to the Average Light x Pct light data in the analysis, but was somewhat weaker in the relation to industry and people activity, and we therefore excluded this version of the light data from the analysis. (Additional information about this light data analysis is available from the authors upon request.) The pixel (light) gain values range from 0 to 63 for the Saturated Light, while the radiance data contains light values that range from 0 to 846 for Sweden (and higher in other parts of the world).

### Swedish Data

The empirical and analytical novelty of the work reported on here results from the use of geo-coded population and establishment counts for Sweden, and its spatial matching with light-emissions data. The Swedish data is compiled by Statistics Sweden [26] and covers all individuals and all establishments in the country. Since each individual and establishment use a unique identification number in relation to tax authorities, governmental bodies, etc. data collected from these different Swedish authorities is combined into one unified dataset by Statistics Sweden. When the data is made public, all observations are anonymized. This data can be accessed only from Sweden, under the Personal Data Act. Statistics Sweden offers access to researchers via secure access to the special retrieval system Microdata Online Access (MONA).

The geo-coded socio-economic data is divided into square grids; the size of these squares depends on whether the specific territory is classified as urban or rural. Urban squares have an area of 250 by 250 meters (1/16^th^ square kilometer), while the rural squares have a size of 1,000 by 1,000 meters (1 square kilometer). To our knowledge, no other country provides data on both total population (night-time residential data) and all establishments (day-time industrial data) at such a fine-grained spatial resolution, and it is this resolution that makes the data suitable for matching with the light-emission data. Statistics Sweden categorizes each place as urban or rural based on population density. In order to be defined as urban, the place should have at least 200 inhabitants, and the distance between the houses cannot be more than 200 meters. There are a number of exceptions allowed from the 200 meters rule, e.g., if the area is disconnected by sports arenas, cemeteries, parks, roads or parking lots. In the 2010 count of the Swedish population, approximately 85 percent of the total population was living in urban areas. Whether a square is urban or rural is defined by the neighborhood level of “urbanity,” rather than the regional or metropolitan definition. This implies that a metropolitan region can have both urban and rural squares, depending on the level of urbanity in each specific square. In the present discussion we utilize two kinds of definitions: count data and density data. In the case of density, we calculate this measure based on the area of the actual square.

We have access to data on the squares where all Swedish individuals reside (in other words, a geo-coding of the total population) as well as the square in which they work (provided they have a job), which means that we can take both day- and nighttime activities into account. We use data on the number of individuals and their total wage income, as well as the density of the two. Wage income is in this context defined as night-time (take-home) wages (in other words, the income individuals bring home to where they live). The constructed variables are based on where individuals reside, in other words, the nighttime population and their wage income. We also use data on counts of establishments in order to measure economic activity in each of the squares. We think that number of establishments—physical locations which presumably use artificial lighting—is a fairly straightforward way to connect economic activity with the generation of NTL data. It is however important to remember that wage is supposed to be more related to productivity levels, and not capture production values as such (measured as gross regional product). Information is available, on a per square basis, on the number of establishments, the number of employees in these establishments, and the total wage bill paid by the establishments (in other words, where the incomes are earned).

In addition to counts we calculated density measures (dividing by land area) which, from a population perspective, reflect their daytime activities. Based on research by e.g. Elvidge et al. 1997 [6]; Townsend and Bruce 2010 [15]; Doll et al 2000 [16]; Letu et al 2010 [24], we would assume the NTL to also be related to energy use. Unfortunately, no energy use data would be available at this level of analysis. The only related data would be CO2 consumption, but this data is only available at a much more aggregated regional level for Sweden, and only for very few years. However, if we correlate CO2 consumption at this level with residential and industry data for Sweden, we find stronger results for industry related variables than for residential based variables. Since the population is distributed across a larger geographical area than the establishments, the population dataset covers 188,986 squares while the establishment data covers 115,496 squares.

**Fig 1:**
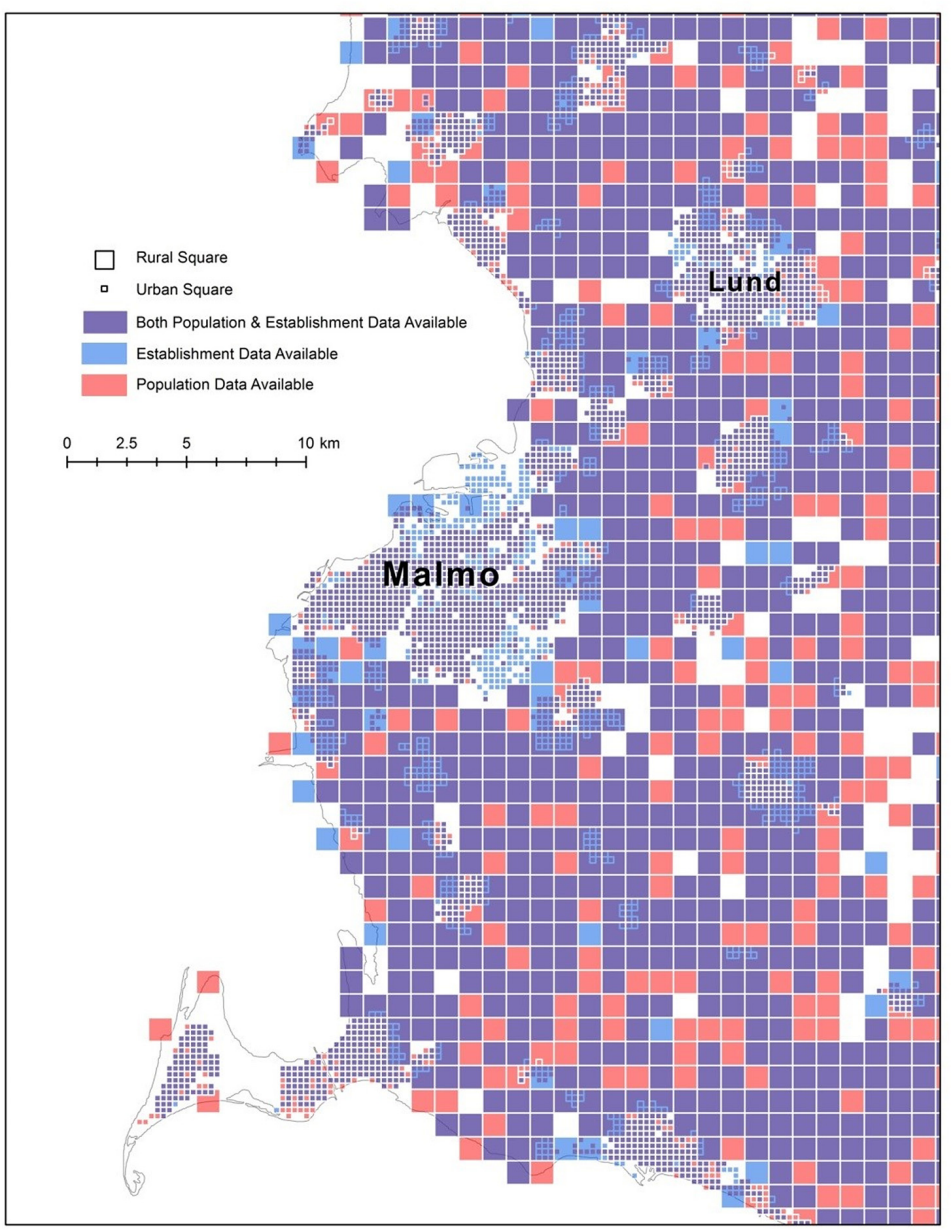
The variation in coverage of the population and establishment data. (Map produced in ArcGIS based on data from Statistics Sweden).

Fig 1 illustrates the variation in coverage of the population and establishment data using the Malmo area as an example. The difference between rural (1,000 by 1,000 meters) and urban (250 by 250 meters) squares is also illustrated in this figure.

All values in the analysis are for the year 2006 so that we can compare results using the standard and radiance NTL data. It would have been preferable to run the analysis over several time periods (monthly or yearly) but given our data availability our analysis is a cross-section.

### Matching the NTL and Socio-Economic Data

To compare the light data with the Statistics Sweden demographic data we generated a grid based on coordinates provided by Statistics Sweden with their demographic data, as described in the section above. The light data was then summarized to this grid to allow for analysis. Using ESRI’s ArcGIS software we created a grid of squares based on the lower left coordinates provided by Statistics Sweden. Separate grids were generated for both industry and population data, resulting in two grids overall (the coordinates provided were based on the data available). The *squares* making up each grid are 250m x 250m in urban areas and 1,000m x 1,000m in rural areas (defined by Statistics Sweden).

**Fig 2:**
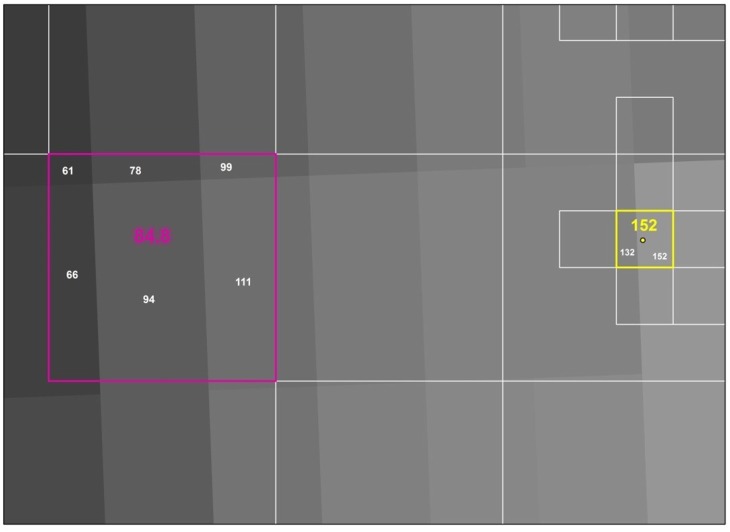
The generation of the average light value per square (Map produced in ArcGIS based DMSP/OLS data).

The methodology used to aggregate the light data to the squares depended on the size of the square (urban vs. rural) because of how they compared to the size of the cells in the light grid. The TIFF raster image was converted to integer format to obtain the light (pixel) values and complete these calculations. Note that we did not clip the image to the Sweden boundary until after these calculations because that would have resulted in no data for areas that should have a light value. To calculate the light values for the rural squares we used the “Zonal Statistics as Table” tool in ArcGIS. This tool is used to summarize raster values within a polygon (the rural squares). We used the mean statistic and so the raster value generated for each rural square was the average light value from all the cells that intersected a square. The pink square in Fig 2 illustrates how this mean value was obtained.

The white numbers within the square are the pixel values for each cell of the light data. 84.8 (in pink) is the average of these 6 values and as a result, is the pixel value assigned to that square. The same process is applied to every rural square across Sweden. The zonal statistics as table tool was less effective for the urban squares due to their small size. Because most of the urban squares only covered part of a single cell (with some exceptions) we used the “Extract Values to points” tool. This tool generates a light value for the centroid of each square by taking the value of the cell that the point is on. This is illustrated in the yellow square in Fig 2. Although the square contains pixel values for two different cells, the center of that square lands on the cell with a value of 152 and so that square is assigned a pixel value of 152. Once light values for each square were generated, we joined this data back to the Statistics Sweden demographic data to complete the analysis.

### Variables

The following are the variables geo-coded for Sweden and used in the analysis. (Summary statistics are presented in Table 1.)

**Table 1:** Data Descriptives (Note: 10 SEK is approximately equivalent to 1.5 USD).

Variable	N	Min	Max	Mean	Std. Deviation
**Light** *					
Radiance	188,986	.00	846.00	50.03	91.81
Saturated	188,986	.00	63.00	17.16	19.96
**Population**					
Total Population	188,986	1	1,696	23.30	52.12
Population Density (per km^2^)	188,986	1	27,136	310.50	844.44
Wage Incomes (100 SEK)	188,986	0	4,481,690	55,293	131,138
Wage Income Density (100 SEK per km^2^)	188,986	0	71,707,040	750,219	2,118,500
**Light** **					
Radiance	115,496	.00	846.00	75.31	114.25
Saturated	115,496	.00	63.00	23.35	22.01
**Establishments**					
No of Establishments	115,496	1	451	3.94	10.14
No of Employees	115,496	0	14,577	32.79	167.02
Wage Sums (100 SEK)	115,496	0	48,882,297	82,350	547,740
Establishment Density (per km^2^)	115,496	1	7,216	50.94	163.91
Employee Density (per km^2^)	115,496	0	233,232	483.47	2,611.53
Wage Sum Density 100 (SEK per km^2^)	115,496	0	782,116,752	1,239,033	85,72,241

*Descriptives for light emissions for the squares with people (N = 188,986).

**Descriptives for light emissions for the squares with establishments (N = 115,496).

#### Population

This is the total employed population (workers) in each square. This is the night-time population value. We also use an employed population density value, which is equal to the total population per square kilometer in each square.

#### Wage Incomes

This is the total wage sum in each square. This value is counted based on the square where the person earning this income lives, in other words, night-time wage income value. We also use wage income density, based on the wage income of the night time population, per square kilometer.

#### Establishments

This is the total number of establishments in each square. We also employ an establishment density value, which is equal to the total number of establishments per square kilometer.

#### Employees

We use the total number of employees in each square, as well as the employee density values. This is equivalent to the daytime, employed population.

#### Wage Sums

This is the wage sums paid by the establishments in each square, which to a certain degree would be related to the aggregated productivity of the establishments in each square. Also in this case, we employ both count data as well as a density measure.

As can be seen in Table 1, we divide the dataset based on either employed population or establishments. Since the employed population is distributed across a larger geographical area, we have more squares for this data set (188,986). In order to have information about a square, at least one person needs to be living in that place. The average number of employed population in each square is 23.3, and the average population density per square is 310. As for wage incomes, the average total wage income per square is 55,293 SEK (approx. 8,400 USD) and the average wage income density is 750,219 (SEK per km^2^, approx. 114,000 USD). For the establishment data, the average number of establishments in each square is 3.94 and the average number of employees per square is 32.79. The wage sums paid by these establishments are on average 82,350 SEK (approx. 12,500 USD), and the density is 1,239,033 SEK per km^2^ (approx. 188,000 USD). In each square, we find on average 33 employees, and the employment density is on average 51 employees per km^2^.

## Empirical Results

### Correlation Analysis

We now turn to the empirical analysis where we connect the light-emission data with the population and establishment data across the squares. We begin with a basic correlation analysis so as to identify bivariate relations between light-intensity and the activity in the same geographical places. We employ both Radiance light as well as Saturated Light in the correlations, to be able to examine possible differences. (The correlation between radiance and saturated light is approximately 0.92.)The results are presented in Table 2. We also ran correlations for specific types of industry and occupational structures, with all correlation coefficients being significantly weaker than for total people or industry values (the results are available from the authors upon request).

**Table 2:** Correlation Analysis.

Variables	Radiance		Saturated Light	
	Totals	Density	Totals	Density
*People*				
No. of People	.597**	.763**	.530**	.725**
Wage Incomes	.524**	.700**	.475**	.666**
*Establishment*				
No. of Est.	.490**	.757**	.399**	.719**
No. of Emp.	.475**	.679**	.410**	.636**
Wage Sums	.456**	.542**	.424**	.512**

**indicates significance at the 1 percent level.

The correlation between people and light is slightly stronger when we use the radiance light-emissions instead of the saturated light. However, the overall structure is very similar, with the strongest correlations between density and light. The correlation between light and the number of people is 0.597 (for radiance) vs. 0.530 (for saturated light), which could be compared with the correlation between light and people density (0.763 vs. 0.725). The correlation between light and wage income is also stronger for the density of wages (0.700 vs. 0.666) compared to total wage income correlations (0.524 vs. 0.475). In other words, we find light to be a better estimation of population than wage incomes. Also, light seems to be capturing density better than the actual number of people or the total amount of wage incomes.

For the establishment correlations, we find similar correlation structures between light and the establishment variables, regardless of whether we use the radiance or saturated light data. However, we find slightly stronger relations between these variables and the radiance light data, compared to the saturated light correlation coefficients. In general, the density variables are again slightly stronger than the count variables which indicates that dense economic activity produces more light than the number of establishments or number of employees. We find the strongest correlations between light emissions and establishment density (0.757 or 0.719 depending on if we use radiance or saturated light). The same correlations for the actual number of establishments are 0.490 vs. 0.399. Also employment density is more strongly related to light emissions than the number of employees (0.679 vs. 0.636 to be compared to 0.475 vs. 0.410). While light has been used as a measure of productivity, out of the three industry variables, light-emissions seem to be the least related to wage sums paid by the companies in each square. The correlation with wage density is equal to 0.542 vs. 0.512 while the correlation with total wage sums is 0.456 vs. 0.424.

### Regression Analysis

Since we would expect that light, as well as people and establishment activities, would spill over across squares of such a small size as the ones we employ here, we now continue to a regression analysis where we’re able to take such effects into account. We ran Geographically Weighted Regressions (GWR), which we then compared with OLS regressions. While the single OLS regression results add very little compared to the bivariate correlation analysis, we use it as a baseline for comparison reasons when we run the geographically weighted regressions, and we also use them to generate Moran’s I values. GWR is a technique which allows us to examine possible spatial non-stationarity, by the use of distance-weighted sub-samples of the data. This implies that we can produce locally linear regression estimates for every point in space, in other words, we generate *β*, which is a local estimate, for every observation. This also means that if the estimated relations were systematically affected by higher latitude regions up in the northern parts of Sweden, the effect would be captured by the individual β coefficients generated in these regions and visible in the maps generated from the analysis. The methodology makes it possible to compare the unstandardized *β* coefficients from the OLS estimation (the global estimate) with the locally produced GWR unstandardized *β* coefficients, to see if those are significantly above or below our “globally” estimated unstandardized *β* coefficient. In other words, the GWR estimation produces information about parameter variation over space (for more detailed information about GWR estimations see Brunsdon et al. [27]). The difference between GWR and spatial autocorrelation techniques is that the latter identify spatial dependence through the residual, while GWR addresses spatial non-stationarity directly through the estimated parameters. In a GWR we assume the regression model to be:

$$y_{i}=\beta_{0}\left(i\right)+\beta_{1}\left(i\right)x_{1i}+\beta_{2}\left(i\right)x_{2i}+\ldots +\beta_{n}\left(i\right)x_{ni}+\varepsilon_{i}$$ (1)

with the estimator

$$\beta^{\prime }\left(i\right)={\left(X^{T}W\left(i\right)X\right)}^{-1}X^{T}W\left(i\right)Y.$$ (2)

where W(i) is a weight specific matrix to location i, so that observations near to i are given greater weight than distant observations.

However, in our empirical analysis below, all estimations will be in single regressions due to the strong multicollinearity between the independent variables. In other words, *y* is our dependent light variable, and *x* is either a population based or industry based socio-economic variable in a single regression. The aim is still to examine if we find any strong geographic interdependencies between the squares, in other words, to compare the OLS generated β-values with the GWR generated ones. All GWR has been produced using Arc Map software. Given that the radiance emissions generated somewhat stronger correlation coefficients, we will use the radiance light-emission (instead of the saturated light data) as dependent variable in all regressions below. All regressions are run in a log-log functional form.

In all regressions, the GWR generated AIC value is below the equivalent value from the OLS regression, indicating that we improved the model and results by allowing variations across space. Moran’s I values generated from the OLS regression also suggested that spatial autocorrelation was present at the 1 percent level, using 100, 200 and 400 neighbors. (We had to restrict our tests to 400 neighbors, since that was the maximum number of neighbors in some cases, given that we geographically do not cover all space in Sweden, but only places with individuals or establishments.) For the GWR estimations we used an adaptive version, which identify the optimal adaptive number of neighbors. The results suggest that the spatial relation is relatively local still. The number of neighbors is below 100 in all cases except the total wage incomes, wage income density, and wage sum density regressions. Given that the squares are of a relatively small size, the actual distance across which we experience spatial relations is quite small. Out of the variables we employ in our estimations, wage density (both people and establishment based) experience the largest spatial effect in the estimations of the unstandardized *β* coefficients.

The variables with the lowest explanatory value, with the most neighbors and with the smallest difference between the OLS and GWR AIC values are people wages, both total wages as well as wage density. These results are also in line with the earlier correlation analysis (see Table 3) where industry wage and industry wage density had among the weakest correlation coefficients.

**Table 3:** OLS and GWR Regression Results (Dependent Variable: Radiance).

	Min	Lower quartile	Mean	Global (OLS)	Upper quartile	Max	Neighbors	R2	OLS AICc	GWR AICc	Residual Squares	No of Observations
*People*												
Total Population	-0.616	0.009	0.101	0.724	0.147	1.522	62	0.356	649,648.9	198,778	26,167.99	115,496
Population Density	-0.366	0.055	0.172	0.544	0.261	0.963	116	0.582	568,130.7	239,680	35,784.33	115,496
Total Wage Incomes	-0.075	0.090	0.170	0.424	0.226	0.908	407	0.275	672,166.9	459,727.3	122,551.2	115,496
Wage Income Density	-0.022	0.165	0.231	0.400	0.294	0.599	639	0.490	605,692.4	453,967.4	120,111.8	115,496
*Establishments*												
No of Establishments	-2.685	-0.010	0.088	0.944	0.115	3.649	30	0.240	417,341.5	98,928.66	10,370.47	188,986
Establishment Density	-0.612	0.032	0.171	0.692	0.257	1.814	62	0.573	350,702.4	115,807	15,311.45	188,986
No of Employees	-0.695	-0.002	0.045	0.420	0.060	1.684	38	0.226	419,521	113,875.5	13,074.59	188,986
Employment Density	-0.241	0.014	0.091	0.417	0.136	1.168	69	0.461	377,607.3	141,256.6	19,401.43	188,986
Total Wage Sums	-0.270	0.001	0.031	0.176	0.045	0.495	87	0.208	422,154.3	185,909.5	29,480.34	188,986
Wage Sum Density	-0.057	0.010	0.040	0.171	0.059	0.398	112	0.294	408,902.2	198,244.6	33,932.07	188,986

All regressions are in log-log format.

**Fig 3:**
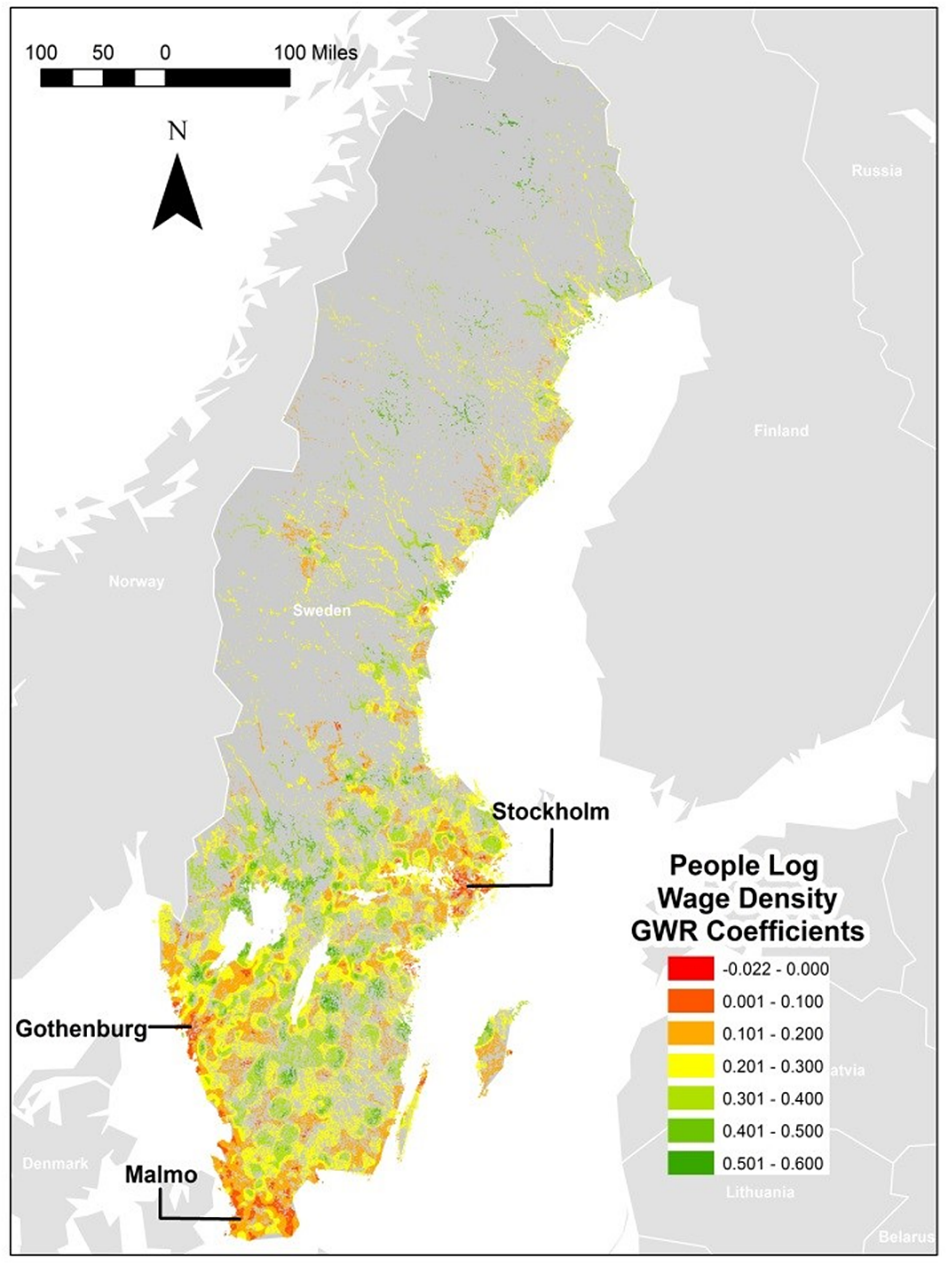
Geographically Weighted Regression Coefficients for People Wage Density. (Map produced in ArcGIS based on data from Statistics Sweden).

**Fig 4:**
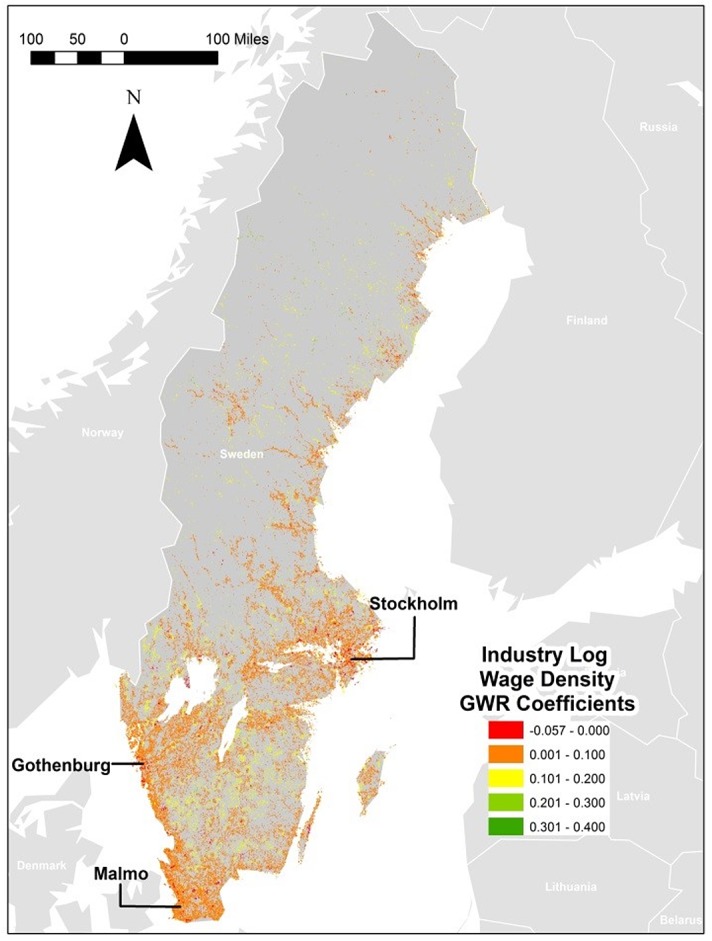
Geographically Weighted Regression Coefficients for Establishment Wage Density. (Map produced in ArcGIS based on data from Statistics Sweden).

If we examine the generated *β* coefficients from our analysis, we can see that the OLS generated global coefficient seems to be an over-estimation for most squares. In all cases, the GWR generated estimates are below the global one up to the upper quartile. In order to examine if there are any clustering effects for those residuals, we produced a map based on each GWR regression. Fig 3 illustrates the GWR estimations for people and Fig 4 illustrates establishment wage density, which we will use to illustrate spatial differences. All additional maps related to each of the GWR in Table 3 are shown in the Supporting information (S1 File). These maps illustrate the relation between light and total population ((Figure A in S1 File); population density (Figure B in S1 File); total wages (Figure C in S1 File); total no of establishments (Figure D in S1 File); establishment density (Figure E in S1 File); total no of employees (Figure E in S1 File); employment density (Figure F in S1 File); and total wage sums (Figure G in S1 File). However, they suggest that there are little spatial clustering effects in those cases. We are aware of earlier work pointing at the possible spatial autocorrelation also for geographically weighted regressions, see e.g. Leung et al., [28] but we are unable to complete such tests because it was computationally prohibitive given the large data set employed in the study.

Figs 3 and 4 clearly illustrates the difference in clustering effects for the individually estimated *β* -coefficients from the geographically weighted regressions. While the value of the *β* coefficients are more or less equally distributed across the squares in the establishment wage density regression (map to the right),we notice a minor over-valuation in the bigger regions, such as Stockholm and Gothenburg, which are marked out in red. When we turn to the results for the geographically weighted regression where people wage density explains light (the map to the left), we see how the individually estimated *β* -coefficients from the regression have a very different, and more clustered pattern. The regions with *β* coefficients closest to the globally estimated one (based on the OLS regression) are the larger cities of Stockholm, Gothenburg and Malmö, indicated by the orange areas in the map. In the darker blue and turquoise areas, the globally estimated *β* -coefficient is an underestimation. These are basically sparsely populated areas or small city regions.

When we examined the maps from all other equations, we found patterns very similar to the establishment wage density regression map, with very little clustering for the individually estimated *β* coefficients. We found bigger cities to somewhat over-estimate the *β* value while the rest of the country (medium sized and smaller cities, as well as sparsely populated areas) tend to underestimate the *β* values.

## Discussion

We have examined the relationship between night-time light (NTL) emissions and several variables capturing economic activity. While much research has focused on the use of night-time light as a proxy variable for urbanization and growth, the question of what exactly artificial light emissions are capturing has been left largely unexamined at a micro-level. While we are not looking at the exact mechanism for light-emissions and how the satellite would measure those emissions, we are looking at a much finer spatial level with both (day-time) industrial and (night-time) residential data. By matching NTL data to the fine-grained, geo-coded micro data for Sweden, we have been able to assess just how closely NTL data tracks the presence and activities of wage earners and establishments.

Our results suggest that night-time light is a somewhat better proxy for night-time population than day-time establishment density, and it is best used as a proxy for density rather than total values, and can thereby indirectly act as a measure of urbanization. However, NTL is relatively weakly related to economic activity as measured by total wages, which in theory are closely related to productivity. With correlations of approximately 0.5, we conclude that NTL emissions are a relatively weaker proxy for economic activity at a micro-level. We examined radiance (non-saturated) light, as well as saturated light, and found that radiance light had a somewhat closer connection to economic activity when compared to saturated light. Further, we found that NTL captures density better than total count values, and that the correlation coefficients in general increased to approximately 0.7, when we compared to people and establishment density. This suggests that night-time light may be a better proxy for the degree of urbanization, as captured by density, than total population, total number of establishments, or total production or consumption in terms of wages. These results also suggest that the saturated light from the annual data series must be used with care.

The results obtained from estimating geographically weighted regressions also suggest that there is spatial dependence in the estimations of economic activity using night-time light, and that these estimated values depend on the results of the neighbor squares. The relationship between night-time light and economic activity, both in terms of people and establishments, varies across regions, primarily based on regional size. This connection tends to be overestimated, especially for wages, in bigger urban regions, while it is underestimated in small and medium sized regions as well as in rural areas.

It should be noted that prior work has relied on some kind of algorithm to generate local estimates of GDP using national GDP, population, or other independent measures to develop a way to calibrate the economic activity estimates for each unit of NTL reported. These results offer additional support for following that type of approach but offer two important caveats. First, since the underlying relationship between NTL readings and local economic activity is not as strong as the relationship with population and population density, the estimates from algorithmic adjustments made will be of limited value or will be such that the adjustments rather than the underlying NTL data is driving those estimates. Second, our results clearly show that the saturation problem is quite real. Any estimates or algorithmic processes that adjust the estimates without taking saturation into consideration are likely to be biased and would underestimate the activity associated with larger, brighter cities and regions.

A very pertinent question, more a caveat perhaps, is the extent to which the results presented here have any bearing on the use of NTL data to study urbanization in developing areas of the world. Sweden might indeed be a good proxy for OECD economies but most assuredly not for African or Latin American ones. But if NTL emissions are not closely tied to economic activity in an advanced economy, why should they be in less advanced settings? Confirming that NTL emissions are or are not closely correlated with economic activity in developing economies will require its own investigation, but note the catch-22 here: it is the very difficulty of measuring economic activity in developing areas, and in particular in urban areas of the developing world, that motivates the use of NTL emissions as a proxy measure in the first place. On a more positive note, the findings that NTL emissions are best used as a proxy for population and establishment density, as opposed to wages, surely suggests that it is a more suitable dataset to be used in developing regions where population is expanding (and concomitantly, so is the informal economy).

There is a pressing need to measure and compare socio-economic activities at a truly global scale. Night time lights data has come to be seen as a very good indicator of urban expansion and distinctly urban activities. Here we have shown that NTL emissions are also correlated with economic activity at the ground level although it is a relatively stronger measure of population density than wages-generating activities. Thus the use of NTL data to construct measures of aggregate economic output need to be undertaken with caution and with attention to the structure of the economy which is being purportedly measured. Ultimately, NTL generation is a function of wealth, but past certain levels of income it is may not be reasonable to expect that more of the earned income would be used in light-generating activities.

## Supporting Information

S1 Fig People–Total Population. (TIF)

S2 Fig People–Population Density. (TIF)

S3 Fig People–Total Wage Incomes. (TIF)

S4 Fig Establishments–Number of Establishments. (TIF)

S5 Fig Establishments–Establishment Density. (TIF)

S6 Fig Establishments–Number of Employees. (TIF)

S7 Fig Establishments–Employment Density. (TIF)

S8 Fig Establishments–Total Wage Sums. (TIF)

S1 File Maps generated from the geographically weighted regression presented in Table 3. (All supporting information maps produced in ArcGIS based on data from Statistics Sweden).
(DOCX)
