# Supplementary figures and images for: Night-Time Light Data: A Good Proxy Measure for Economic Activity?
Source: PLoS One. 2015 Oct 23;10(10):e0139779. doi: 10.1371/journal.pone.0139779 (PMC4619681; doi:10.1371/journal.pone.0139779)

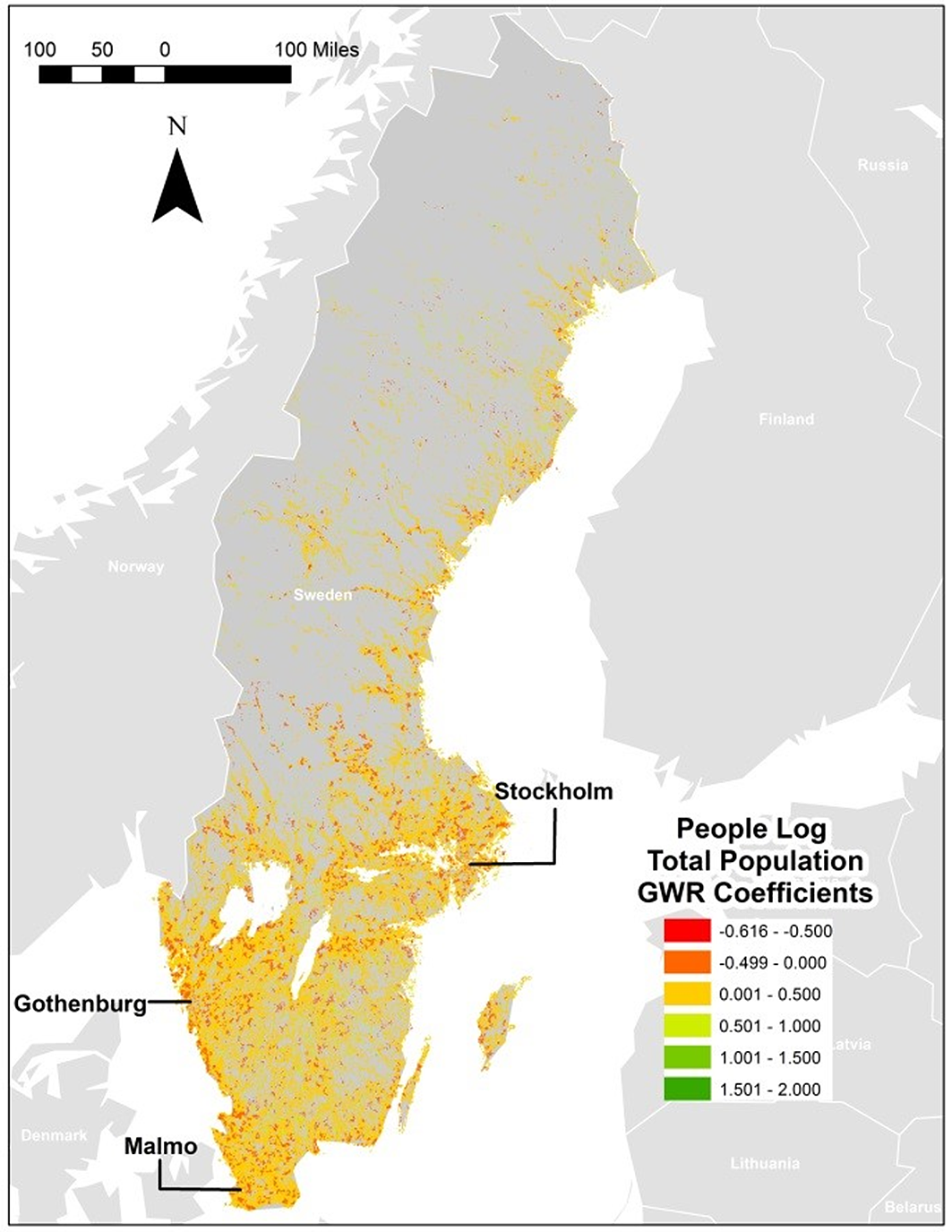

Supplement: S1 Fig — (TIF) [file pone.0139779.s001.tif]

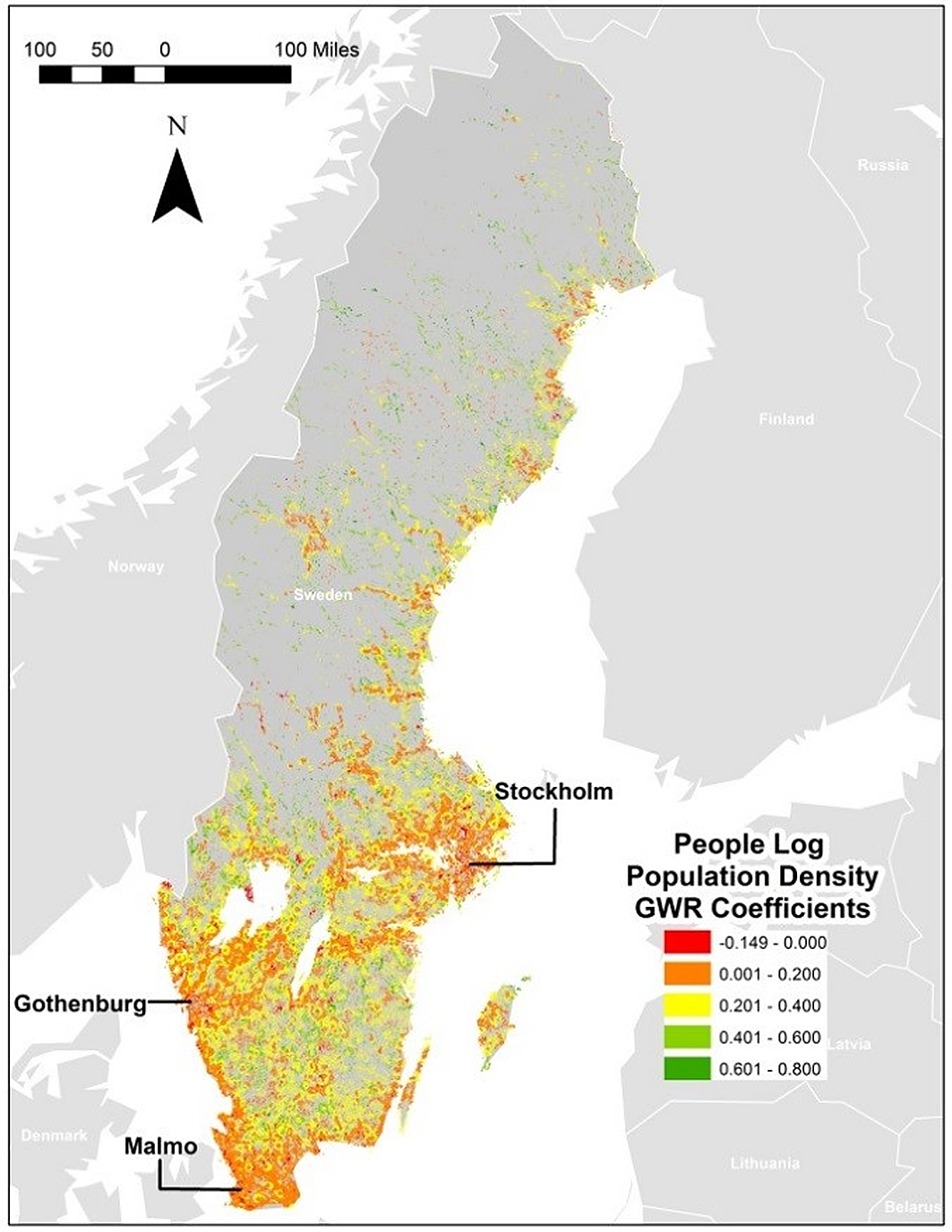

Supplement: S2 Fig — (TIF) [file pone.0139779.s002.tif]

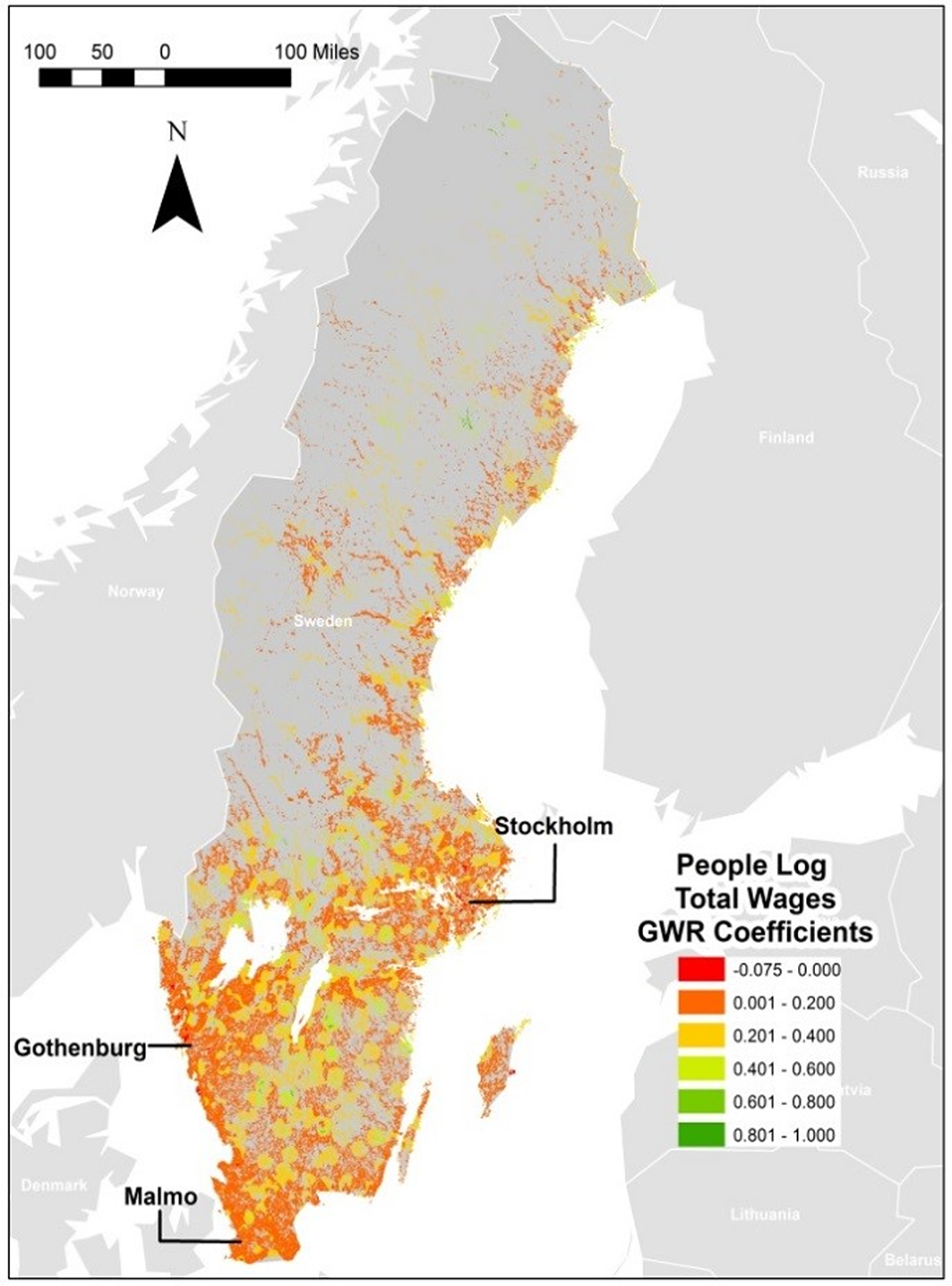

Supplement: S3 Fig — (TIF) [file pone.0139779.s003.tif]

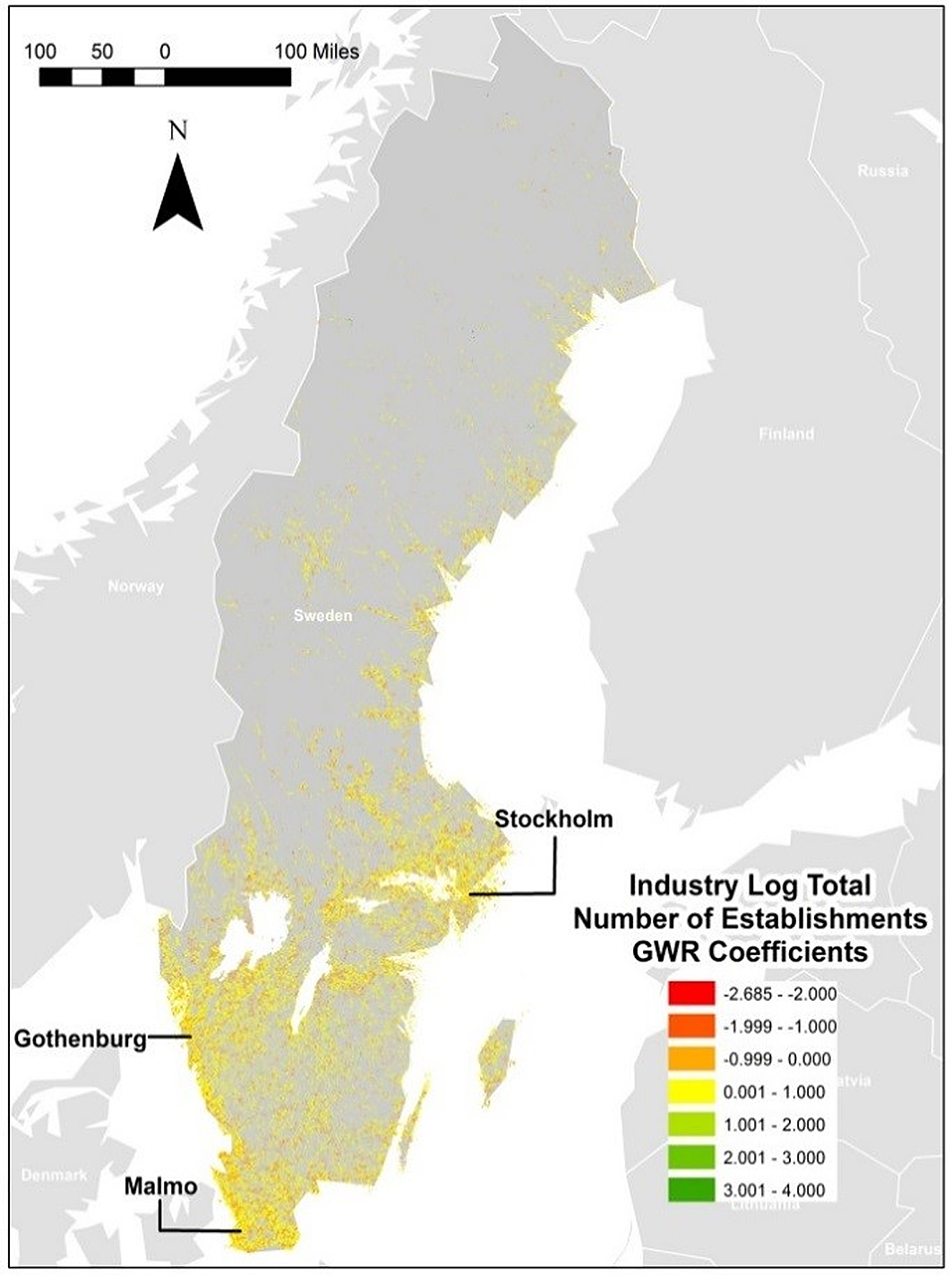

Supplement: S4 Fig — (TIF) [file pone.0139779.s004.tif]

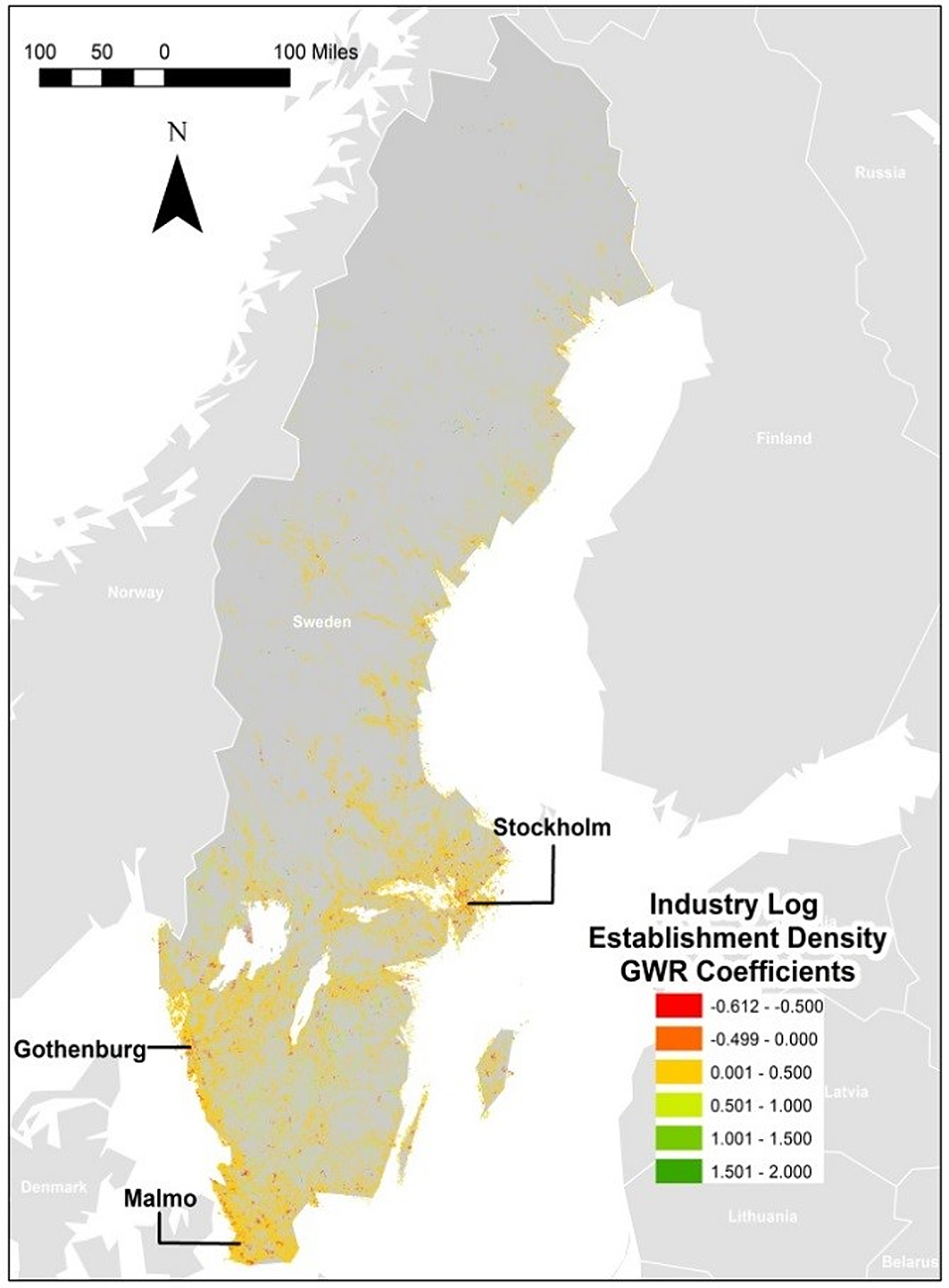

Supplement: S5 Fig — (TIF) [file pone.0139779.s005.tif]

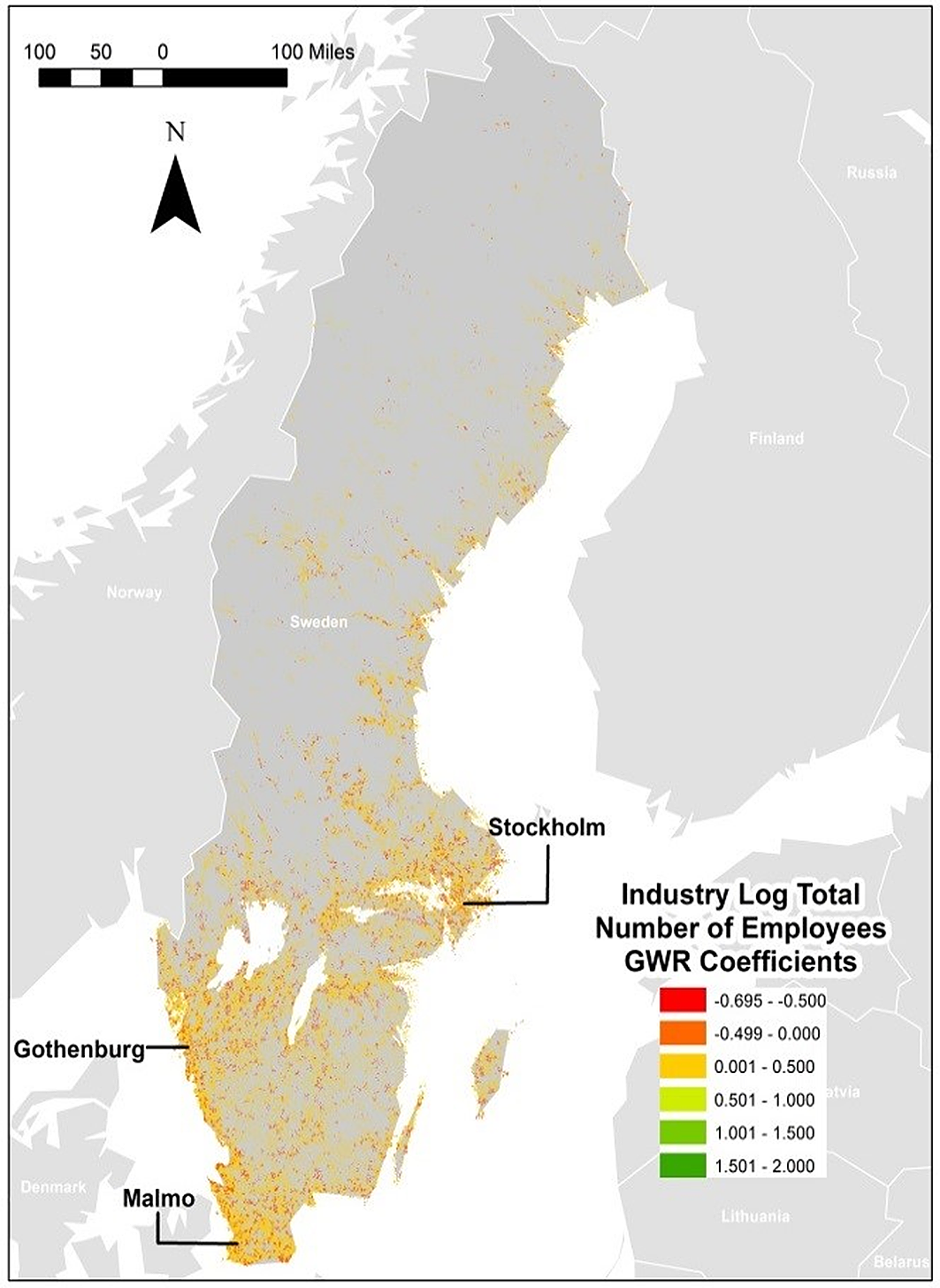

Supplement: S6 Fig — (TIF) [file pone.0139779.s006.tif]

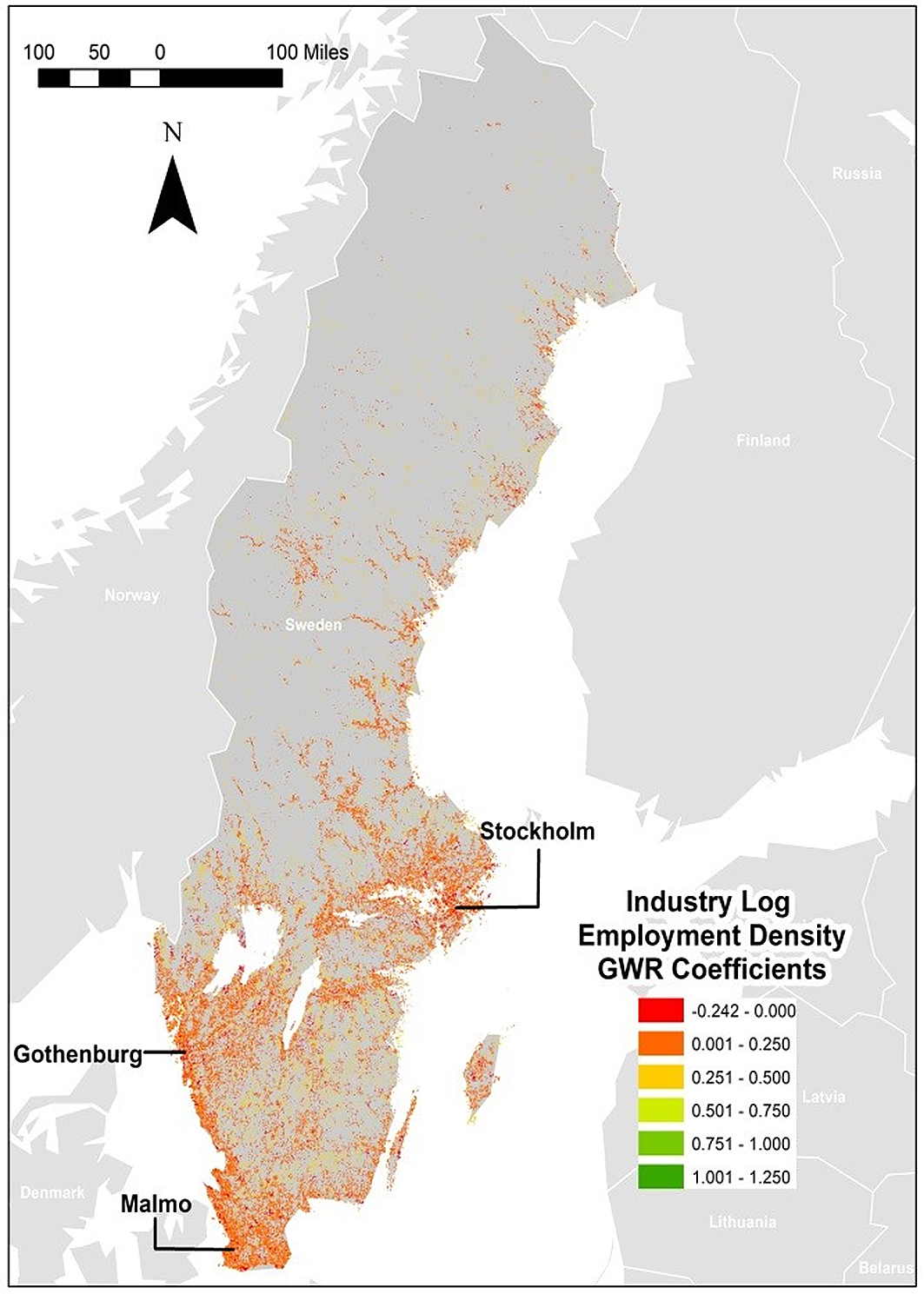

Supplement: S7 Fig — (TIF) [file pone.0139779.s007.tif]

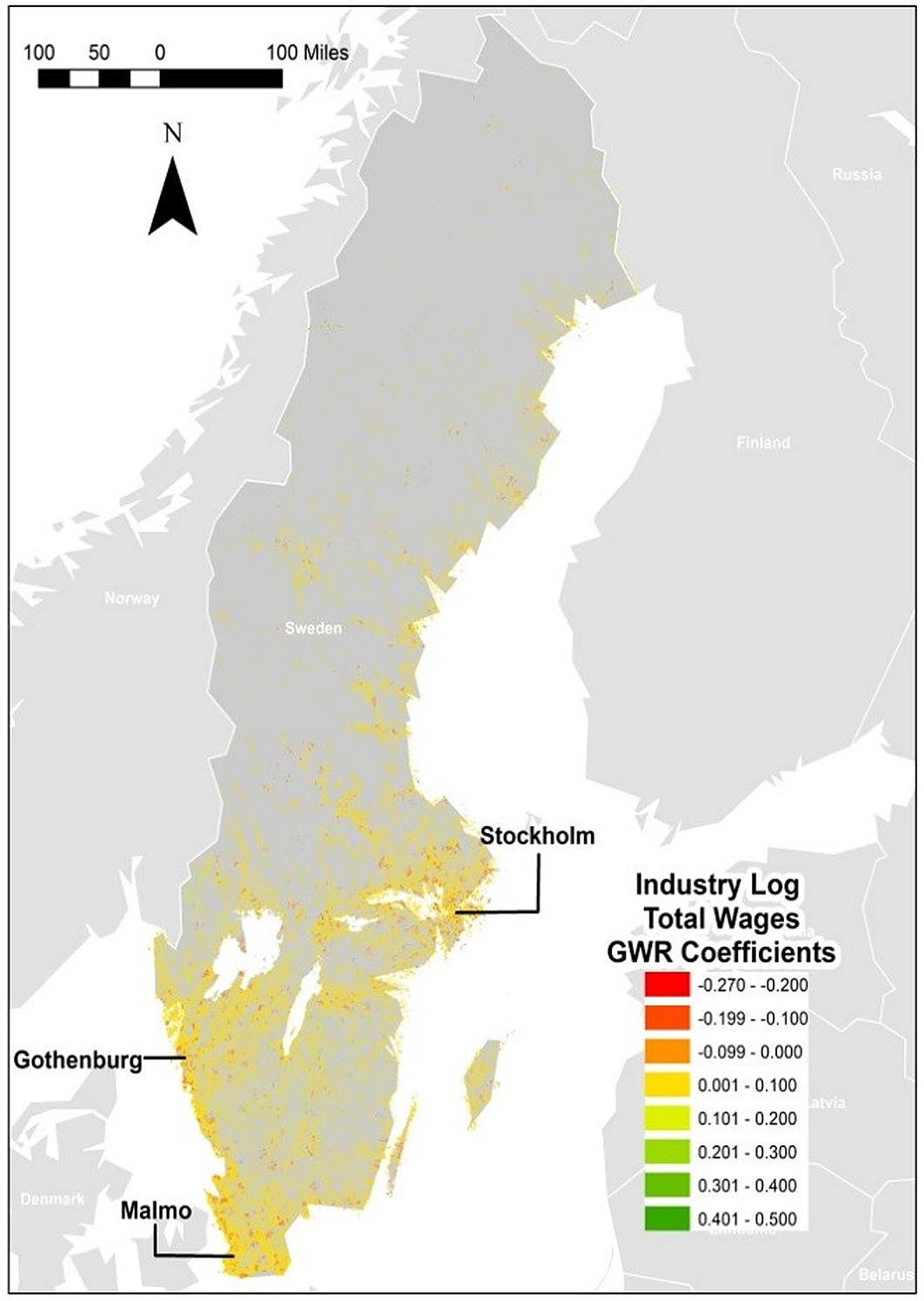

Supplement: S8 Fig — (TIF) [file pone.0139779.s008.tif]
