# Supplementary material for: Night-Time Light Data: A Good Proxy Measure for Economic Activity?
Source: PLoS One. 2015 Oct 23;10(10):e0139779. doi: 10.1371/journal.pone.0139779 (PMC4619681; doi:10.1371/journal.pone.0139779)
Supplement: S1 File — (All supporting information maps produced in ArcGIS based on data from Statistics Sweden). (DOCX) [file pone.0139779.s009.docx]

Supporting Information: GWR MAPS (All supporting information maps produced in ArcGIS based on data from Statistics Sweden)

| **(S1 Fig about here)** | **(S2 Fig about here)** |
| --- | --- |
| 1. S1 Fig: People - Total Population | 1. S2 Fig: People - Population Density |

| **(S3 Fig about here)** | **(S4 Fig about here)** |
| --- | --- |
| 1. S3 Fig: People – Total Wage Incomes | 1. S4 Fig: Establishment – Number of Establishments |

| **(S5 Fig about here)** | **(S6 Fig about here)** |
| --- | --- |
| 1. S5 Fig: Establishment –Establishments Density | 1. S6 Fig: Establishment – Number of Employees |

| **(S7 Fig about here)** | **(S8 Fig about here)** |
| --- | --- |
| 1. S7 Fig: Establishment – Employment Density | 1. S8 Fig: Establishment – Total Wage Sums |
